# Barriers and enablers to community living: A mixed-methods systems study of young adults with life-limiting conditions

**DOI:** 10.1177/02692163261418445

**Published:** 2026-02-22

**Authors:** Ali Lakhani, Deniz Senyel, Ruth Mackenzie-Stewart, Rwth Stuckey

**Affiliations:** 1 School of Psychology and Public Health, La Trobe University, Melbourne, VIC, Australia; 2 The Hopkins Centre, Menzies Health Institute Queensland, Griffith University, Meadowbrook, QLD, Australia

**Keywords:** palliative care, home care services, informal caregivers, qualitative research, health services accessibility, social support, systems analysis

## Abstract

**Background:**

Adults aged 18–64 years with life-limiting conditions often fall through gaps in health and disability systems, particularly due to service ineligibility. Although home-based palliative care improves outcomes and cost-effectiveness, structural inequities and fragmented services can prevent younger adults from remaining in the community.

**Aim:**

To identify factors influencing the ability of younger adults with life-limiting conditions to remain in the community and to map how these factors interact – using Australia as an illustrative context – through a systems-thinking approach.

**Design:**

Mixed-methods study combining retrospective case-note analysis and semi-structured interviews. Thematic coding informed a causal loop diagram visualizing system dynamics and leverage points.

**Setting/participants:**

Sixty-three younger adults connected to a national care-coordination program supporting those at risk of residential aged-care entry due to ineligibility for disability or aged-care funding. Data comprised 48 case notes and 15 interviews across all Australian jurisdictions.

**Results:**

Five interdependent domains influenced community living: informal care, formal care, service navigation, respite access, and condition severity. The causal loop diagram revealed three reinforcing and two balancing loops. Key leverage points included respite access, carer wellbeing, and streamlined navigation and eligibility processes.

**Conclusions:**

Systems thinking clarified how interactions among informal care, formal supports, and symptom severity shape care continuity. Findings highlight the need to position respite, practical carer support and navigation assistance as core components of community-based palliative care, and to align disability, aged-care and palliative-care systems so younger adults are not excluded from essential support due to age or funding rules.

**What is already known about the topic?**

- Community-based palliative care enables people with life-limiting conditions to die at home but remains under-resourced and fragmented.
- In high-income countries, younger adults with life-limiting conditions are frequently underserved due to misalignment between disability and aged care systems.
- Informal caregivers play a pivotal role in sustaining home-based care, yet their needs are often overlooked.

**What this paper adds?**

- Five interdependent domains are identified in this study—informal care, formal care, respite access, condition severity, and system navigation—as central to remaining in the community.
- Three reinforcing and two balancing feedback loops that govern care continuity for younger adults with life-limiting conditions are presented in a causal loop diagram.
- Navigation ability and carer wellbeing emerged as modifiable leverage points that influence service access and home-based care sustainability.

**Implications for practice, theory or policy**

- Targeting respite availability and caregiver support can initiate positive feedback loops that reduce premature institutionalization.
- Integrating systems thinking into palliative care research can reveal structural barriers and guide system-level policy change.
- Policy reform across comparable health and disability systems is needed to harmonize eligibility criteria and provide timely, coordinated support for younger adults with life-limiting conditions.

## Introduction

Most terminally ill patients prefer to die at home,^1,2^ among the closest to them and cared for by their general practitioner.^3–5^ Home-based care cuts hospital use^6^ and is cost-effective.^7^ Community based palliative care (a mix of home-based clinical, informal/unpaid care provided by family, friends and/or people in the community^8,9^) and formal supports (paid care by health/social care professionals) – can assist to ensure some patients remain at home throughout their palliative journey, including end-of-life.^10,11^ Systematic reviews^5,12–14^ identify clinical status, preferences and local supports determine home death.

## Challenges in providing home-based palliative care

Home-based palliative care remains under-resourced,^15^ while a lack of (i) alignment of national standards with home-based programs, (ii) sustainable financing, and (iii) awareness of existing support resources^16,17^ provide further barriers. Informal carers face heavy physical, emotional and social burdens,^18^ and supporting their needs, through training and practical supports, is crucial for enabling care at home.^19,20^ The burdens faced by informal carers impact their physical and mental health.^21–23^ Expanded formal care can ease that burden.^24^ For palliative care practice, this means that supporting carers and coordinating non-clinical help are central to whether home-based care can be sustained.

Globally, approaching 60% of adults who require palliative care are under the age of 65, and many experience considerable significant barriers to accessing services at home.^25^ When these supports are unavailable or difficult to navigate, younger adults may be directed toward institutional settings such as long-term care or residential aged-care facilities, not because of clinical need but because community alternatives are lacking.^10–12^ This pattern has been described across multiple countries, including Canada,^26^ the United States,^27^ the United Kingdom,^28^ and Australia.^29^ Within these settings younger adults report feeling socially isolated, disconnected from peers, and poorly matched to environments primarily designed for older populations.^27–30^ These experiences frequently include a loss of independence, limited opportunities for age-appropriate social participation, and negative impacts on mental health.^28–32^ Institutional environments can also expose younger adults to physical, psychological and social harm due to unsuitable care models and insufficient resourcing.^30^ These challenges are often compounded by fragmented funding streams and policy gaps that limit access to alternatives to institutional accommodation.^33^

### Context for the current study

In this paper, we use the term “younger adults” to refer to people aged 18–64 years with life-limiting conditions. Internationally, younger adults with life-limiting conditions often fall through gaps between health, disability and aged-care systems, where age-based eligibility rules and fragmented service pathways limit access to coordinated support.^27–30^ Australia provides one example of these broader structural issues. Younger adults with life-limiting conditions in Australia are often ineligible for the National Disability Insurance Scheme – (Australia’s first federal self-directed approach to health and social service provision for people with disability) – unless they demonstrate they meet functional capacity impacts required by National Disability Insurance Scheme guidelines including their practitioners verifying surgical/medical interventions have been fully explored.^34^ These integration gaps, observed internationally, also manifest in Australia, where inadequate coordination between health, disability, and aged-care sectors limits support for younger adults with life-limiting conditions.^35^

Coordinating stakeholders and tailoring support plans, the program helps younger adults with life-limiting conditions remain in their communities. Examining the experiences of participants, both those diverted from aged care and not, clarifies barriers and enablers of home-based care and informs strategies to personalize support. Consequently, this study addressed the following questions:

- What are the experiences for younger adults (and/ or their families) with life-limiting conditions who are not eligible for federal funding and who are at risk of entering residential aged care? Specifically, what are the barriers for younger adults (and/ or their families) that prevent them from remaining in the community, and what enablers support these younger adults remaining in the community?
- How do diverse facilitators and barriers interact in a complex system to impact the ability for people with life-limiting conditions to remain in the community?

By examining these questions, the study aims to inform both palliative care practice – through clearer understanding of what enables home-based support – and policy, by identifying system changes needed to reduce potentially avoidable admissions to residential aged care.

## Methodology

*Design*: A qualitatively driven mixed-methods systems study was undertaken, combining retrospective review of case notes and summary reports with semi-structured interviews with clients and informal carers connected to the Younger People in Residential Aged Care System Coordinator Program. The study followed a convergent design^36^ in which case-note and interview data were analyzed thematically and then integrated through the development of a causal loop diagram. Mixing occurred primarily at the levels of analysis and interpretation: themes from each data source were brought together in a shared coding framework and then synthesized to identify system-level feedback loops and leverage points.

*Setting*: The study was conducted within the Younger People in Residential Aged Care System Coordinator Program, a national care-coordination initiative delivered by Ability First Australia. The program operates across all Australian states and territories and supports younger adults at risk of entering residential aged care because they are ineligible for disability or aged-care funding packages.

*Sampling*: A purposive sampling strategy was employed for both case-note data and interviews. “Diverted clients” are defined as younger adults who were supported to avoid admission to residential aged care, whereas “non-diverted clients” are those who were not able to be diverted from residential aged care. For the case-note component, Ability First Australia provided de-identified case notes and summary reports for all “diverted” clients (those supported to avoid admission to residential aged care; *n* = 13) and a random selection of “non-diverted” clients (those who could not be diverted from residential aged care; *n* = 36) stratified by state or territory, with five non-diverted cases per jurisdiction where available. Sample size was driven by both feasibility and depth rather than formal power calculations, as the analysis was primarily qualitative and descriptive. Emphasis was on achieving thematic consistency across case notes and interviews. For the interview component, all eligible current or former clients and carers who had previously consented to be contacted for research were invited to participate.

*Recruitment*: Ability First Australia emailed 140 eligible clients and informal carers who had provided prior consent to be approached for research participation. Interested individuals contacted the research team directly, received an information sheet, and were re-consented prior to interview. Participation was voluntary, and no financial incentives were offered. Recruitment continued until data sufficiency was reached, with a final sample of 15 interviews (14 client or carer interviews, plus one case in which both case-note and interview data were available).

*Data collection*: The deidentified retrospective case note and summary reports review captured demographics and service contacts. Additional demographic client information: Gender, Age at Referral, and Aboriginal and/or Torres Strait Islander status, was shared by Ability First Australia. The semi-structured interviews (~1 h) were held via Zoom or phone. These explored participants’ experiences of barriers and facilitators to remaining in the community and the impact of the Younger People in Residential Aged Care System Coordinator Program program on their health and wellbeing. Semi-structured interviews were conducted by the project leads, AL and RS, both senior academics with extensive experience in qualitative health research and in interviewing people with disability, life-limiting conditions, and informal carers. An interview guide was used to ensure consistency across interviews, and the team held regular debriefing meetings to maintain a uniform interviewing approach and reflexive practice throughout the study. Interviews were audio-recorded and transcribed verbatim. This information was stored securely on La Trobe University’s Lab Archives platform, with each transcription labeled by a unique participant identifier.

*Data Analysis*: Themes were first generated from the retrospective case-note review, and an initial codebook was developed. This codebook was then deductively applied to interview transcripts. Codes were derived from the study questions, the case-note review, and the Systems Approach to Healthcare Delivery framework,^37^ and were organized into three domains:

1. Barriers and facilitators to remaining in the community
  - client factors (symptom control, cognition, social-care needs, location)
  - care-team factors (availability, timeliness, navigation of formal/informal care)
  - system factors (housing, service access, policy constraints)
2. Client experience with the Ability First Australia program
  - service navigation, first contact, interactions with hospitals/social workers, overall satisfaction
3. Client wants, needs, and service gaps
  - shortfalls of in-home support, alternatives to aged-care facilities, funding limits

Coding was managed in NVivo. Two researchers synthesized case-note, summary-report and interview findings into a causal loop diagram (using Vensim^38^), iteratively mapping feedback relationships to depict how these factors interact to influence younger adults’ ability to stay in the community. During this process, thematic saturation was evident, as no new concepts were emerging across the data sources, supporting confidence in the completeness of the coded themes.

Mixed-methods integration occurred in three ways. First, we connected the data sources by using insights from the case-note review to refine the interview guide and the codebook applied to interview transcripts. Second, we merged themes from case notes and interviews within the shared coding framework, allowing convergent and divergent perspectives to be compared across data sources. Third, we built on these integrated themes to construct a causal loop diagram using Vensim,^38^ iteratively mapping directional relationships and feedback loops across client, carer and system-level factors. A causal loop diagram, a systems thinking tool, was used to map the relationships among various factors contributing to a problem.^39^ Coded data was distilled into key factors and directional links derived. Each link is tagged “+” when the variables move together and “–” when they move in opposite directions.^40^ Through this process, we identified reinforcing and balancing feedback loops and potential leverage points for improving community living outcomes for younger adults with life-limiting conditions.

*Ethical Considerations*: This study was approved by the La Trobe University Human Research Ethics Committee (Reference: HEC23228). For the case-note component, only de-identified data were provided to the research team. For the interview component, participants received written and verbal information about the study and provided written consent prior to participation. Participants were reminded that involvement was voluntary, that they could decline to answer any question. All data were stored securely on password-protected institutional servers, accessible only to the research team.

## Findings

*Participants*: The demographics of the 63 participants (15 interviews and 48 case-notes) have been included within Table 1.

**Table 1.** Participant characteristics.

Characteristic	Number
Diverted from aged care	
Diverted	13
Not diverted	50
Data source	
Case notes	48
Case notes and interviewed	1
Interviewed	14
Gender	
Female	31
Male	32
Aboriginal Torres Strait Islander status	
No	51
Unknown	12
State/Territory	
Australian Capital Territory	1
New South Wales	13
Northern Territory	5
Queensland	9
South Australia	9
Tasmania	6
Victoria	14
Western Australia	6
Regional classification	
Major City	33
Inner Regional	17
Outer Regional	12
Remote	1

The distribution of participants across States, Territories, and remoteness categories does not reflect Australia’s population proportions.^41^ Regional classifications follow the Australian Statistical Geography Standard,^42^ where “Major City,” “Inner Regional,” “Outer Regional,” and “Remote” indicate differing levels of population density and access to services.

## Informal care in the home

Informal caregiving by family and friends emerged as integral to enabling young people with life-limiting conditions to stay at home. This was identified as important to helping clients remain at home in 47.6% of all case notes (*n* = 30) and 100% of diverted clients. Participants called it a round-the-clock job: *“He wouldn’t be able to remain in the community if someone is not here with him 24/seven. I feel very sad for all those people out there who don’t have an advocate, who don’t have anyone that they can turn to in their circumstances.”* (ID03).

## Navigating health and social services

Caregivers stressed that home care succeeds only when they can navigate services: *“. . .my job then became to make sure that I covered all the bases . . . to find out if there was any service available at all. That was a lot of legwork, because there’s lots of services there. . . . . . . .gaining adequate and appropriate in-home support and care relies on an advocate who is willing to just turn over every stone.”* (ID06). Others reported unsuccessfully searching “*endlessly*” for help. Friction with providers added strain: *“I’ve been organising everything for my husband and then as soon as he goes into hospital and these other professionals get on it, you’re treated like a frigging dill. . . . . Like you know nothing. You’re not part of the conversation.”* (ID02).

## Health and wellbeing of informal carers

Caregivers described severe fatigue and scant respite. One said, *“With cancer, in particular, you have to look after the carer as well as the person with the disease.”* (ID05) Sustaining counseling proved hard: *“I started with a counsellor, but I’ve got to be honest – she was lovely, but I just found that it was such – it did help, but it felt like it was a time constraint.”* (ID08) The emotional toll was profound: *“It’s consumed my time and it’s consumed my life and sometimes I just – I know I shouldn’t say this. Sometimes I want my bloody life back.”* (ID09) This impact could be long-term: *“So that went on for a year. So I had carer’s burnout for a long time. Very long time.”* (ID11), highlighting the ongoing need for emotional support, respite opportunities, and carer resources.

## Respite

Respite mattered but was provided rigidly; the care received was mostly appreciated but there were issues with inflexibility: *“you can have this on Monday at 2 o’clock and if it doesn’t suit you, too bad, so sad.”* (ID06), and inadequate availability: *“I chose someone - a company to come down and give me and my son respite. All that paperwork and everything finally went through, they came down on the Tuesday so that we could go into town for five hours.”* (ID02).

## Formal care in the home

Both groups relied on formal aid, but diverted clients more so: 62% receiving income support (mainly Disability Support Pension), one-third accessed in-home palliative care, and nearly half obtained domestic-care packages. Only 10% of non-diverted clients secured comparable funding, some tapping superannuation instead. Formal care service gaps remained clear – *“There is nothing available on weekends. It’s nine to five, Monday to Friday.”* (ID10) – and options were narrow: *“they said, oh, do you need help with showering? Well no, I don’t. [Client] and I are managing with showering. Oh, well that’s all they can offer.”* (ID02) Rigid scheduling frustrated families, while occasionally over-servicing felt intrusive: *“I was getting the phone calls while he was in hospital to say that we’re getting support workers and they’re going to be there eight hours a day and for seven days a week and I - and I had to go, no, that’s way too much. I could not stand to have people in the house for that time factor.”*

## Clients condition and situation

Barriers to staying home spanned complex illness, functional loss, poor housing and, at times, refusal of help. Case-note data identified 62% of diverted clients reported serious medical or situational hurdles – cancer, neurological deficits, unsafe housing, partner illness – while six resisted assistance. Non-diverted cases showed similar patterns: chronic pain, falls, incontinence, and oxygen need or tracheotomy-care affected 16%, with frequent hospital stays. Interview data reinforced these findings, particularly regarding cognitive decline, which increased caregiver burden. As one caregiver described: *“He used to get very forgetful. He’d go into the kitchen to put the stove on—because they had gas—and he’d walk out. He’d forget it was on.”* (ID14) Falls were another key concern: *“She’s got the cancer in her hip, and if she falls over, that’s the end of her.”* (ID09) For some, illness progression led to complete dependence, increasing care demands: *“He was pretty much getting to be bedridden and needed that 24-hour care.”* (ID01) Progressive conditions and cognitive decline quickly eclipse informal capacity, making responsive formal support essential.

## Ineligibility or unavailability of services

Eligibility barriers blocked care across the cohort. Most diverted clients (62%) reported National Disability Insurance Scheme refusal, slow end-of-life services, lack of suitable housing or oxygen-use limits; one-quarter of non-diverted clients faced similar gaps – National Disability Insurance Scheme denials, scarce palliative or hospice options, and inadequate home support. Some avoided applying, expecting systemic delays and challenges: *“We kept getting told that you’re too old for NDIS and too young for My Aged Care. . .you’re the wrong age group.”* (ID09), while another: *“I’ve now found out I’m not eligible for NDIS either because I am quote, ‘diseased, not disabled.’”* (ID05) With no alternative, families resorted to aged care, underlining the need for flexible eligibility rules and timely, integrated support.

## Causal loop diagram clarifying factors contributing to remaining in the community

**Figure 1.**
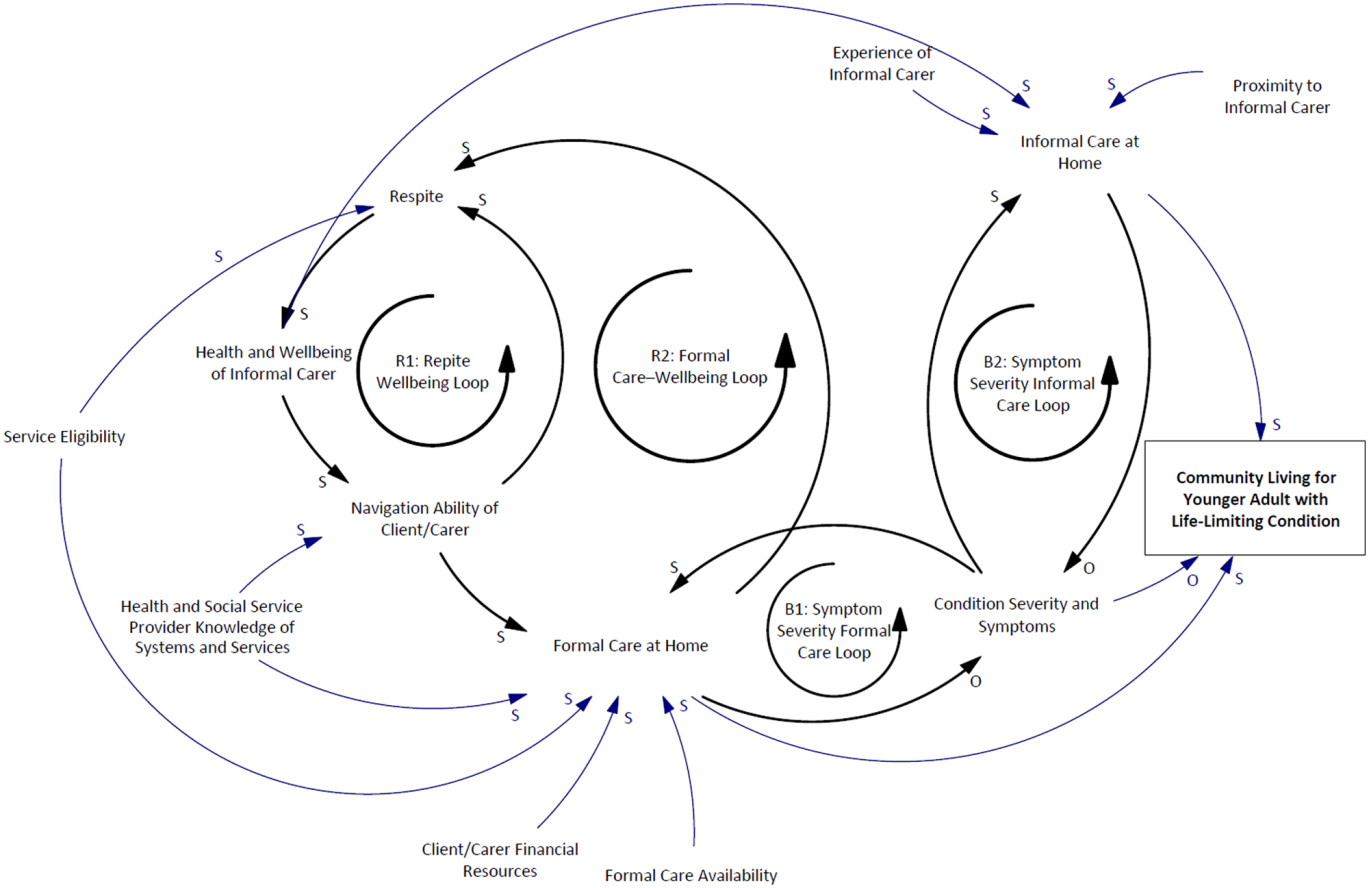
Causal loop diagram illustrating factors influencing the ability of younger adults with life-limiting conditions to remain in the community.

Figure 1 is the causal loop diagram derived from case notes and interview data. It highlights five key feedback loops shaping community living outcomes for younger adults with life-limiting conditions:

- R1: Respite–Wellbeing Loop – Access to respite improves carer wellbeing, enhancing their capacity to navigate systems and secure ongoing support.
- R2: Formal Care–Wellbeing Loop – Formal care supports carer wellbeing and strengthens navigation ability, reinforcing access to continued formal care.
- R3: Experience–Informal Care Loop – As carers gain experience, their capacity to deliver effective informal care increases, promoting stability.
- B1: Symptom Severity–Formal Care Loop – Increasing symptoms prompt formal care, which may help reduce severity and restore balance.
- B2: Symptom Severity–Informal Care Loop – Similarly, greater symptom severity leads to more informal care, which can also mitigate decline.

The diagram also maps the influence of service eligibility, financial resources, provider and carer system knowledge, and proximity to care, on the availability and quality of both formal and informal care.

## Discussion

Policies integrating non-clinical support within palliative care and the broader healthcare system are essential to ensure equity, access and continuity of care. The World Health Assembly’s resolution on palliative care highlights this need for integration of health care settings, including community-based care.^43^ Integration is crucial to facilitating palliative care services throughout clients’ illness trajectory,^44^ supported by comprehensive national strategies facilitating effective community-based palliative care.^44^ The developed causal loop diagram reinforces this need, identifying respite access and formal care availability as key leverage points to sustaining carer wellbeing and home-based care continuity. For palliative care teams internationally, this implies the need to build stronger links with social care, housing and disability providers, and to routinely assess and respond to carer needs as part of standard care. For policymakers, the findings support investment in funding models that explicitly resource these non-clinical elements of palliative care delivery.

In the Australian context, equity gaps in palliative care arise from system fragmentation, geographic and financial barriers, and broader socioeconomic disadvantage,^45–47^ reflecting patterns also observed in other fragmented health and social care systems.^25^ Our findings support the value of a coordinated home-based support model for this cohort – one that standardizes eligibility, embeds community empowerment in service design, and offers flexible funding for non-clinical supports.

While central to sustaining home care, informal carers remain under-supported, requiring practical education, tangible aids, readily accessed respite, formal recognition in care-plans and access to mental-health services.^48–52^ The causal loop diagram confirms carer wellbeing is not only a direct outcome of service access but also a critical enabler of sustained informal care, therefore a powerful leverage point. Respite, in particular, strengthens wellbeing and system navigation, creating positive reinforcing cycles supporting carers and clients. Hybrid respite facilities offering day, overnight and intermediate stays would be especially valuable for younger adults with intensive needs, reinforcing this cycle of sustainability.

This study has several strengths. It draws on two complementary data sources – retrospective case notes and in-depth interviews – which allowed triangulation. The inclusion of participants from every Australian state and territory provided national coverage and captured a wide range of service contexts, policies, and resource environments. Using a systems thinking approach and developing a causal loop diagram strengthened the analysis by highlighting relationships and feedback patterns that would perhaps not be as apparent through linear or single-method designs.

However, several limitations must be acknowledged. The quality and completeness of case notes varied because they were written by different coordinators, with varying levels of detail. Furthermore, interview participation was voluntary and may reflect the experiences of those more willing or able to engage, potentially under-representing people with higher support needs or limited access. Consistent with qualitative research, the findings reflect the specific contexts studied rather than statistical generalisability. Instead, they offer conceptual and experiential insights into system functioning.

Patient and public involvement was limited to participation through interviews rather than involvement in study design. However, participants were offered the opportunity to review their interview transcripts on request, and a summary of preliminary findings was shared in draft form for clarification and feedback. No participants identified inaccuracies or suggested changes, indicating that their perspectives were appropriately represented. These lived-experience insights directly informed the causal loop diagram and the interpretation of system dynamics.

## Conclusion

This study highlights the complex interplay between formal care, informal caregiving, service navigation, and health status that determines whether younger adults with life-limiting conditions can remain in the community. By using a systems approach and mapping feedback loops through a causal loop diagram, we identified key leverage points – respite access, carer wellbeing, navigation capacity, and service eligibility – that shape care continuity and equity. These findings underscore the need for nationally consistent, flexible, and integrated support systems that empower carers, respond to changing client needs, and reduce reliance on institutional care.
